# Mapping of quantitative trait loci controlling lifespan in the short-lived fish *Nothobranchius furzeri* – a new vertebrate model for age research

**DOI:** 10.1111/j.1474-9726.2011.00780.x

**Published:** 2012-04

**Authors:** Jeanette Kirschner, David Weber, Christina Neuschl, Andre Franke, Marco Böttger, Lea Zielke, Eileen Powalsky, Marco Groth, Dmitry Shagin, Andreas Petzold, Nils Hartmann, Christoph Englert, Gudrun A Brockmann, Matthias Platzer, Alessandro Cellerino, Kathrin Reichwald

**Affiliations:** 1 Genome Analysis, Leibniz Institute for Age Research – Fritz Lipmann InstituteJena, Germany; 2 Faculty of Agriculture and Horticulture, Department of Crop and Animal Sciences, Humboldt-UniversityBerlin, Germany; 3 Institute of Clinical Molecular Biology, Christian-Albrechts-UniversityKiel, Germany; 4 Evrogen JSCMoscow, Russia; 5 Shemyakin and Ovchinnikov Institute of Bioorganic ChemistryMoscow, Russia; 6 Molecular Genetics, Leibniz Institute for Age Research – Fritz Lipmann InstituteJena, Germany; 7 Biology of Aging, Leibniz Institute for Age Research – Fritz Lipmann InstituteJena, Germany; 8 Scuola Normale SuperiorePisa, Italy

**Keywords:** lifespan, *Nothobranchius furzeri*, genetic linkage map, QTL mapping, synteny

## Abstract

The African annual fish *Nothobranchius furzeri* emerged as a new model for age research over recent years. *Nothobranchius furzeri* show an exceptionally short lifespan, age-dependent cognitive/behavioral decline, expression of age-related biomarkers, and susceptibility to lifespan manipulation. In addition, laboratory strains differ largely in lifespan. Here, we set out to study the genetics of lifespan determination. We crossed a short- to a long-lived strain, recorded lifespan, and established polymorphic markers. On the basis of genotypes of 411 marker loci in 404 F_2_ progeny, we built a genetic map comprising 355 markers at an average spacing of 5.5 cM, 22 linkage groups (LGs) and 1965 cM. By combining marker data with lifespan values, we identified one genome-wide highly significant quantitative trait locus (QTL) on LG 9 (*P* < 0.01), which explained 11.3% of the F_2_ lifespan variance, and three suggestive QTLs on LG 11, 14, and 17. We characterized the highly significant QTL by synteny analysis, because a genome sequence of *N. furzeri* was not available. We located the syntenic region on medaka chromosome 5, identified candidate genes, and performed fine mapping, resulting in a *c.* 40% reduction of the initial 95% confidence interval. We show both that lifespan determination in *N. furzeri* is polygenic, and that candidate gene detection is easily feasible by cross-species analysis. Our work provides first results on the way to identify loci controlling lifespan in *N. furzeri* and illustrates the potential of this vertebrate species as a genetic model for age research.

## Introduction

Lifespan is a quantitative trait, which reflects the interplay of genetic and environmental factors and varies largely across animal species. Two interventions have been identified to date in invertebrate, vertebrate, and mammalian model organisms, which consistently extend lifespan: dietary restriction and repression of growth hormone/insulin-like growth factor 1/insulin receptor pathways. However, the underlying molecular mechanisms remain unclear (e.g., Kirkwood, 2008), and current knowledge of genetic players is largely based on the analysis of single-gene mutations. An unbiased approach to identify genetic factors controlling lifespan would ideally consist of the analysis of genotype–phenotype associations in crosses of inbred strains that largely differ in the trait. This approach is named quantitative trait locus (QTL) mapping and offers also the possibility to identify natural alleles relevant for trait variation. A number of lifespan QTLs have been identified in fly and worm (e.g., Shmookler Reis *et al.*, 2006; Lai *et al.*, 2007), yet the detection of underlying gene(s) has proven challenging. Also in the mouse, many lifespan QTLs have been detected (e.g., Lang *et al.*, 2010; Leduc *et al.*, 2010), but crosses are both time-consuming and expensive, and the underlying functional loci have still proven elusive. It would be desirable to identify additional vertebrate/mammalian model organisms, which show larger lifespan differences and a shorter lifespan and could be reared at reasonable cost.

The African annual fish *Nothobranchius furzeri* is a promising candidate model for such QTL studies. It is a very short-lived vertebrate and shows typical aging-related phenotypes such as physiological/cognitive decay and expression of aging-related biomarkers (Valenzano *et al.*, 2006b). Lifespan and health span can be extended by application of resveratrol and dietary restriction (Valenzano *et al.*, 2006b; Terzibasi *et al.*, 2009). A major advantage of *N. furzeri* as a model to study the genetic architecture of aging is the availability of natural populations and laboratory strains, which differ in lifespan up to 100% (Terzibasi *et al.*, 2008; Hartmann *et al.*, 2009). The lifespan of the model fish species zebrafish and medaka, for which both genome sequences and highly advanced genetic resources are available, is well over 3 years. To our knowledge, naturally occurring zebrafish/medaka strains with significantly different lifespans do not exist.

To facilitate a systematic search for genetic determinants of the *N. furzeri* lifespan, we performed an initial characterization of the *N. furzeri* genome (Reichwald *et al.*, 2009). It is comprised of 19 chromosomes (2n = 38), has a size of 1.6–1.9 giga base pairs (Gb), and is repeat-rich (45%). Of the fish model species with a sequenced genome available (i.e., medaka, stickleback, pufferfish, and zebrafish), medaka was identified as the closest relative showing average protein identity values of 77.4% to *N. furzeri* counterparts. This work contributed also to the first genetic map of *N. furzeri* and to mapping of simple traits such as caudal fin coloration and sex (Valenzano *et al.*, 2009).

Here, we aimed at identifying QTLs influencing the *N. furzeri* lifespan. We performed an F_1_ intercross of the short-lived, inbred strain GRZ (10% survivorship: 10–16 weeks) and the long-lived, recently wild-derived strain MZM-0403 (10% survivorship: 29–31 weeks) (Terzibasi *et al.*, 2008; Hartmann *et al.*, 2009). We identified four QTLs, one of which was highly significant at the genome-wide level. To analyze this QTL in the absence of a genome sequence, we employed cross-species analyses. We defined a list of candidate genes, and by subsequent fine mapping, we reduced the length of the initial 95% confidence interval (CI) 1.6-fold.

## Results

### Experimental crosses

**Figure 1:**
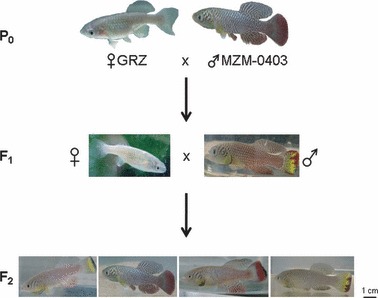
A cross of a short-lived GRZ female and a long-lived MZM-0403 male. The top row depicts the parental generation. The first filial (F_1_) generation (middle) was intercrossed to generate F_2_ progeny (bottom). Allele segregation in the F_2_ generation is exemplified by tail coloration of males.

We crossed GRZ and MZM-0403 in two independent experiments named cross A and cross B. In cross A, a GRZ female was interbred with an MZM-0403 male. From one F_1_ family, 39 F_2_ progeny were obtained. Because this number was not sufficient for our study, we repeated the cross using different fish (cross B). In cross B, we formed six F_1_ families that produced 365 F_2_ offspring, representing the main source of this work (Fig. 1).

### Lifespan of the crossing panel

We recorded the lifespan (age at death) of 334 individually housed F_2_ progeny of cross A and B and performed a combined analysis of the crosses, which we refer to in the following as cross AB. We also collected lifespan data of individually housed specimens of both parental strains (GRZ: *n* = 27 and MZM-0403: *n* = 19), because the previously published data were based on group housing (Valenzano *et al.*, 2006a, b; Terzibasi *et al.*, 2008; Hartmann *et al.*, 2009).

**Figure 2:**
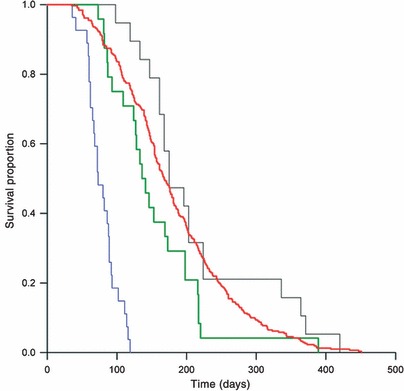
Survivorship of cross AB and GRZ (*n* = 27, blue) and MZM-0403 (*n* = 19, black), as estimated by Kaplan–Meyer analysis. F_1_ progeny (*n* = 24) is given in green; F_2_ (*n* = 310) progeny is shown in red. The mean and maximum lifespans of GRZ and MZM-0403 were significantly different (*P* < 0.001, log-rank test). The F_1_ progeny showed an intermediate and significantly different lifespan compared with both parental strains (*P* < 0.001). The F_2_ offspring had a longer mean and maximum lifespan than GRZ and F_1_ progeny, but less compared with MZM-0403, and was significantly different only from GRZ (*P* < 0.001).

The mean lifespan was 11 weeks (w) for GRZ and 30 w for MZM-0403, and the maximum lifespan values (maximum values always refer to 10% survivorship) were 16 w and 53 w, respectively, which were significantly different (*P* < 0.001, log-rank test). The F_1_ progeny of cross AB showed an intermediate and significantly different lifespan (*P* < 0.001) compared with both of the parental strains (mean, 22 w; maximum, 31 w). The F_2_ offspring of cross AB had a longer mean (26 w) and maximum (43 w) lifespan than both GRZ and F_1_ progeny, but mean and maximum lifespan were shorter than in MZM-0403. The F_2_ lifespan was significantly different from GRZ (*P* < 0.001), but not from MZM-0403 and F_1_ progeny (Table 1, Fig. 2).

**Table 1:** Lifespan data of parental strains GRZ and MZM-0403 and the crossing panel

			Lifespan [days]		
				
Fish	Generation	Number	Mean (SE)	Max	*P*-value†
GRZ	P_0_	27‡	078 (4.2)*	115*	0.321
		12^m^	086 (5.4)	117	
		13^f^	074 (6.8)	122	
MZM-0403	P_0_	19§	213 (21.1)*	372*	0.496
		16^m^	215 (20.3)	375	
		3^f^	244 (91.4)	425	
Cross AB	F_1_	24	154 (14.2)**,***	217**,***	0.263
		10^m^	175 (27.8)	389	
		14^f^	139 (13.7)	216	
	F_2_	310	181 (4.7)**	300**	0.249
		149^m^	174 (6.8)	282	
		161^f^	188 (6.5)	316	

SE, standard error of the mean.

m Males only;

f females only.

† Log-rank statistic between male and female lifespan for each strain and progeny.

‡ 12 males, 13 females, and 2 of unknown sex.

§ 16 males, 3 females.

* Significantly different between parental strains GRZ and MZM-0403 (*P* < 0.001);

** significantly different to GRZ (*P* < 0.001);

*** significantly different to MZM-0403 (*P* = 0.036).

We next analyzed whether there was a sex-based lifespan difference. GRZ and MZM-0403 males showed a mean/maximum lifespan of 12/17 w and 31/54 w, respectively; the data of females were similar. In cross AB, the lifespan of both the male and female F_1_- and F_2_ progeny did not significantly differ as well (Table 1).

We further tested for a correlation of lifespan and coloration pattern, because all GRZ and MZM-0403 males we have observed to date showed a distinct coloration. In the wild, *N. furzeri* males are generally present as ‘yellow’ or ‘red’ morph (Reichard *et al.*, 2009). Yellow morphs show a submarginal yellow and a thin black marginal band in the caudal fin, and red morphs have a broad red marginal band. These two naturally occurring phenotypes appear to be fixed in GRZ (yellow morph) and MZM-0403 (red morph, Fig. 1). In the male F_2_ progeny of cross AB, the lifespan of yellow, red, and intermediate color morphs did not differ significantly (Table S1).

### Genetic characterization of *Nothobranchius furzeri* strains

To provide genetic markers for the GRZ and MZM-0403 strains, we established/genotyped 1253 gene-associated single-nucleotide variations (SNVs; we use variations rather than polymorphisms because we refer to differences between laboratory strains) and 139 microsatellites in up to 20 specimens, respectively.

All of the 1253 SNVs were homozygous in GRZ, whereas 812 (65%) were homozygous and 441 (35%) heterozygous in at least one of the MZM-0403 specimens. Of the 139 microsatellites, we analyzed all in GRZ and 82 in MZM-0403. The average heterozygosity in GRZ was 0.01 (female, 0; male, 0.02), proving that this strain is highly inbred and heterozygous only at sex-linked markers in males (Reichwald *et al.*, 2009; Valenzano *et al.*, 2009). In contrast to GRZ, 99% (81) of the microsatellites were heterozygous in MZM-0403 and showed 2–9 alleles per marker (mean = 6; Fig. S1) with an average heterozygosity of 0.51. In conclusion, the four P_0_ fish used to set up cross A and B, that is, two GRZ females and two MZM-0403 males, genetically represent a trio consisting of one GRZ female and two MZM-0403 males.

### Second-generation genetic linkage map

The first-generation linkage map of *N. furzeri* was composed of 132 microsatellites, forming 25 linkage groups (LGs) with a genetic length of 1012 cM (Valenzano *et al.*, 2009). To provide a map with better resolution and more markers suitable for cross-species analyses, we genotyped 283 gene-associated SNVs and 128 microsatellites in 404 F_2_ progeny of cross AB. The resulting sex-averaged map of *N. furzeri* had 22 LGs with a length of 1969 cM, composed of 355 linked markers at an average intermarker distance of 5.5 cM (Table S2, Fig. S2). On the basis of a maximum intermarker distance of 36 cM, the presence of 13 singletons together with three more LGs than the number of *N. furzeri* chromosomes, we estimated that at least 576 cM (36 × 16) were not accounted for in this map. Thus, the total map length amounted to 2545 cM (1969 + 576). The genome size of *N. furzeri* was estimated between 1.6 and 1.9 Gb (Reichwald *et al.*, 2009); therefore, a genetic distance of 1 cM should be equivalent to 0.63–0.75 Mb in this species.

### Synteny of *Nothobranchius furzeri* and medaka

We next attempted to localize the *N. furzeri* markers forming the current genetic map in the genome of medaka to assess the synteny between both species. We chose medaka, because of the model fish species with a genome sequence available it appeared most closely related to *N. furzeri* (Reichwald *et al.*, 2009).

Most (256 of 355, 72%) *N. furzeri* markers mapped to medaka chromosomes (Chrs). There were unambiguous hits for 208 of 231 (90%) gene-associated markers and for 48 of 124 (39%) microsatellite loci. In more detail, *N. furzeri* LGs 1–18 and 20 showed synteny to medaka Chrs 3–24, with LG 1, 7, 15, and 17 being syntenic to two medaka Chrs, respectively (Fig. S3). In conclusion, the *N. furzeri* LGs 1–18 and 20 most likely represented the core of the 19 *N. furzeri* Chrs.

### Genome-wide search for QTLs affecting lifespan

For the detection of lifespan QTLs, we used cross B (F_2_ progeny: *n* = 284, Table S3) and the genetic map composed of cross AB. We excluded cross A, because there were only few offspring and also because of the heterogeneity of the P_0_ males; that is, the males of cross A and B differed at 39% of the microsatellite markers, which would have complicated the QTL analysis. The lifespan values of cross B and cross AB were similar (Fig. S4 and Fig. 2, respectively).

We performed two types of QTL analysis, a first one including all F_2_ progeny and a second one including a dataset, in which we had eliminated the first 25% of deaths, that is, excluding all fish prior to 122 days of age (methods). This procedure is common in mouse lifespan QTL studies; it eliminates the effects of early (age-independent) mortality (e.g., Jackson *et al.*, 2002) and was empirically proven to improve the detection of longevity QTLs (Klebanov *et al.*, 2001).

When analyzing all F_2_ progeny, we found that lifespan was correlated with maximal body weight (detailed in statistical analyses). In the respective first QTL scan, QTLs were detected on LG 1, 9, and 13 (Fig. S5). The LG 1 QTL showed genome-wide significance (*P* < 0.05), whereas the QTLs on LG 9 and 13 were suggestive (LG-wise significant at *P* < 0.05). However, most of the markers within the 95% CI of the LG 1 QTL showed a sex bias in allele distribution: one allele from the P_0_ male was present almost exclusively in F_2_ males (Data S1). This was consistent with a genetic sex-determination system, in which males represent the heterogametic sex, as described for *N. furzeri* (Valenzano *et al.*, 2009). The LG 1 QTL overlapped with the reported sex-determining region, making it impossible to separate sex-specific from lifespan-related effects. Separate QTL scans of males/females did not identify QTLs, most likely because the number of progeny was not sufficient (data not shown).

**Figure 3:**
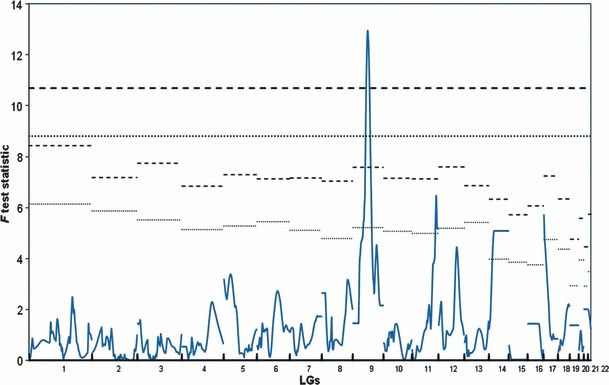
*Nothobranchius furzeri* lifespan QTLs. *F*-test statistic values are given on the *Y*-axis; linkage groups (LGs) are depicted on the *X*-axis. The space allotted to a respective LG reflects the relative genetic length. Continuous dashed and dotted lines show empirically determined *F*-value thresholds for genome-wide highly significant (*P* = 0.01) and significant (*P* = 0.05) linkage. Interrupted lines represent LG-wise highly significant (*P* = 0.01) and significant (*P* = 0.05) linkage. QTL, quantitative trait locus.

The ranked lifespan data of the F_2_ progeny, excluding 25% of early deaths, was not correlated with body weight (statistical analyses). The second QTL scan revealed four QTLs influencing lifespan, which were located on LG 9, 11, 14, and 17 (Fig. 3, Table 2). The highest effect on lifespan was estimated at a peak *F*-value of 12.96 at 46 cM on LG 9 near gene-associated marker *SUCLG2* (GTP-specific beta subunit of succinate-CoA ligase). This QTL was highly significant at the genome-wide level (*P* < 0.01) and had the same location as in the first QTL analysis. The QTLs on LG 11, 14, and 17 were suggestive (Table S4) showing highest *F*-values near microsatellite marker Nfu_0030_FLI on LG 11, gene-associated markers *EF1*a (eukaryotic translation elongation factor 1 alpha 1) and *APOE* (apolipoprotein E) on LG 14, and *SFT2D1* (SFT2 domain containing 1) on LG 17 (Table 2).

**Table 2:** Most likely positions and effects of QTLs

LG	QTL [cM]*	95% CI†	Length of CI [cM]	Marker‡	*F*-value	Additive (SE)§	Dominance (SE)§	% Variance¶
9	46	29.5–74.0	44.5	*SUCLG2* (47.2)	12.95	0.389 (0.140)	−0.830 (0.191)	11.3
11	75	0.0–76.0	76.0	Nfu_0030_FLI (75.1)	6.47	0.465 (0.133)	−0.154 (0.179)	6.0
14	58	5.5–60.0	54.5	*EF1A* and *APOE* (59.2)	5.09	0.483 (0.311)	−2.647 (1.059)	4.8
17	0	0.0–30.5	30.5	*SFT2D1* (0.0)	5.72	0.419 (0.125)	−0.068 (0.185)	5.3

QTL, quantitative trait locus; LG, linkage group.

* Estimated QTL position given in centimorgan.

† The 95% confidence interval (CI) estimated by bootstrap analysis.

‡ Marker closest to the LG position with the highest *F*-value; in parenthesis position of marker in centimorgan.

§ Additive (a) and dominance (d) effect and their standard error (SE) determined with ranked, transformed values, the direction given as MZM-0403 allele effect.

¶ F_2_ phenotypic variance.

**Figure 4:**
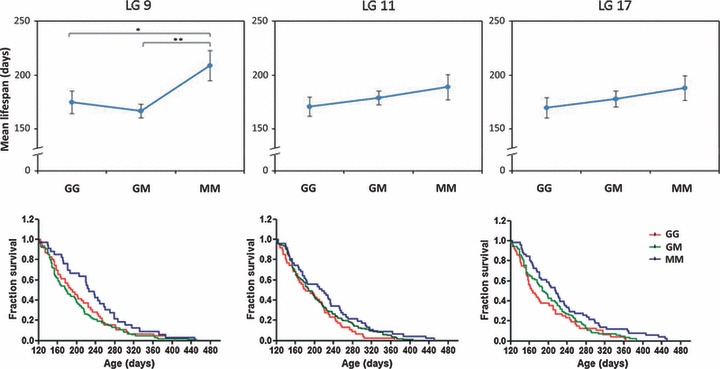
Allelic effects at lifespan QTLs on LG 9, 11, and 17 (top) and survivorship of the F_2_ progeny (below). GG stands for F_2_ homozygous for the GRZ allele; GM refers to heterozygous F_2_ and MM to F_2_ homozygous for the MZM-0403 allele. Mean lifespan and standard errors were calculated for males and females together. Statistical analyses were performed using the *t*-test (GG vs. MM) and the Mann-Whitney test (GM vs. MM and GG). Significant differences are highlighted, one asterisk represents *P* < 0.05, two asterisks represent *P* < 0.01. QTL, quantitative trait locus; LG, linkage group.

At *SUCLG2*, the mean lifespan of the heterozygous F_2_ progeny (in the following referred to as GM for GRZ/MZM-0403) was 167 days compared to 175 days of the F_2_ progeny homozygous for GRZ alleles (GG) and 209 days of the F_2_ progeny homozygous for MZM-0403 alleles (MM). The difference was significant between GM and MM (*P* = 0.004, Mann–Whitney test) and as well between GG and MM (*P* = 0.048, *t*-test) (Fig. 4, Table S5). In addition, the survivorship was significantly different between GM and MM (*P* = 0.017, log-rank test). At the peak markers of the suggestive QTLs, the mean lifespan did not significantly differ between GG, GM, and MM, respectively. However, the survivorship differed significantly between GG and MM at the peak marker of the QTLs on LG 11 (*P* = 0.029, log-rank test) and LG 17 (*P* = 0.012, log-rank test). At these QTLs, there was also a GG < GM < MM trend in survivorship (Fig. 4, Table S5).

The QTLs account for 11.3% (LG 9), 6% (LG 11), 4.8% (LG 14), and 5.3% (LG 17) of the lifespan variance in the F_2_ population, respectively (Table 2). Tests for multiple QTLs were negative (Table S4).

Further, based on the lifespan data, we estimated that 4.7 genes contributed to the trait variation. The heritability of lifespan was 32% in the crossing panel.

### Fine mapping of the highly significant lifespan QTL and targeted mapping of functional candidate genes

**Figure 5:**
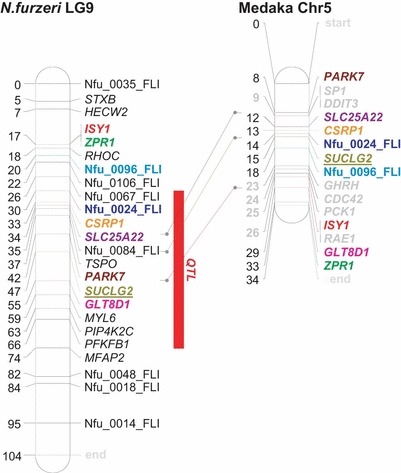
Cross-species analysis of *Nothobranchius furzeri* LG 9 (in cM) and medaka Chr 5 (in Mb). The red bar on the right of LG 9 represents the 95% confidence interval of the highly significant lifespan QTL. Nine markers (colored) of *N. furzeri* LG 9 were assigned to medaka Chr 5, including the peak marker (*SUCLG2*) and flanking genes (*PARK7* and *GLT8D1*). Three colored lines indicate their respective location on the *Nothobranchius furzeri* LG 9 and medaka Chr 5. Six human orthologs of aging-relevant genes (gray) were assigned to this medaka region. Gene names and gene-associated markers are given in italics. Microsatellite markers are given in the following way: Nfu_number_FLI. QTL, quantitative trait locus; LG, linkage group.

The 95% CI of the QTL on LG 9 comprised 44.5 cM, corresponding to 28–33 Mb (Table 2, Fig. 5). If one assumed that the *N. furzeri* genome contains *c.* 20 000 protein-coding genes, *c.* 350 genes were expected to map in this CI. Ten of these genes were known, because they were markers in our genetic map, and six of these could we assign to medaka Chr 5, including the peak marker *SUCLG2* and flanking markers *PARK7* and *GLT8D1* (Fig. 5). The order of *PARK7*, *SUCLG2*, and *GLT8D1* was conserved on medaka Chr 5, but the genomic region encompassed by these genes was twice the size (21.8 Mb) as in *N. furzeri* (13.1 cM corresponding to 8–10 Mb), suggesting that substantial chromosomal rearrangements occurred over evolutionary time between medaka and *N. furzeri*.

We assigned 342 *N. furzeri* transcripts to the interval flanked by *PARK7* and *GLT8D1* on medaka Chr 5 (data not shown). In 25 of these *N. furzeri* transcripts, we established informative markers, the selection being based on a distance of *c.* 1 Mb between these transcripts in the medaka interval. Upon genotyping in cross B, all transcripts were mapped to *N. furzeri* LG 9, thereby increasing the number of markers on the LG to 49. Sixteen of the new markers were located within the original 95% CI of the lifespan QTL (Table S6). In a subsequent genome-wide QTL scan using all LG 9 markers and the F_2_ dataset, in which 25% of early deaths were excluded, we confirmed the highly significant QTL on LG 9 and could reduce the initial 95% CI by a factor of 1.6 (Table S7).

We were also able to assign six human orthologs of known aging-related genes, which were listed in ‘The Ageing Gene Database’ (de Magalhaes & Costa, 2009), to the relevant region on medaka Chr 5: transcription factor Sp1 (*SP1*), DNA-damage-inducible transcript 3 (*DDIT3*), growth-hormone-releasing hormone (*GHRH*), cell division cycle 42 GTP-binding protein (*CDC42*), phosphoenolpyruvate carboxykinase 1 (*PCK1*), and RNA export protein 1 homolog (*RAE1*) (Fig. 5). Following a candidate gene approach, we identified *N. furzeri SP1*, *DDIT3*, *CDC42*, *PCK1*, and *RAE1*, as well as the QTL peak marker *SUCLG2*, and flanking markers *PARK7* and *GLT8D1* by cDNA sequencing. None of the transcripts had a sequence variation in GRZ or MZM-0403 impairing protein function (data not shown).

In a proof of principle experiment for targeted mapping of functional candidates, we analyzed three genes reported to play a role in mammalian aging: *CDC42*, *PCK1*, and *RAE1* (Baker *et al.*, 2006; Hakimi *et al.*, 2007; Wang *et al.*, 2007). In their introns, we established informative markers, showing the genotypes (IVS for ‘intervening sequence’ followed by intron number, intron position of variation, and genotype of strains GRZ/MZM-0403): *CDC42* IVS5 + 119 TT/TC, *PCK1* IVS1 + 30 CC/TT, and *RAE1* IVS4 + 37 CC/TT. Upon genotyping in cross B, we confirmed the predicted localization on *N. furzeri* LG 9 for *PCK1* and *RAE1*, but *CDC42* could not be linked to any LG because of insufficient informativity in the F_1_ families. In detail, *N. furzeri RAE1* mapped at 17.3 cM and *PCK1* at 21.8 cM on LG 9, both being outside of the 95% CI of the lifespan QTL. Therefore, a major impact of these genes on *N. furzeri* lifespan determination seemed rather unlikely.

## Discussion

We present first results of our ongoing efforts to unravel the genetic architecture of lifespan in *N. furzeri*, a new vertebrate model organism for aging research.

### Lifespan of *Nothobranchius furzeri*

We performed individual recordings of single-housed fish. The maximum lifespan of GRZ in single housing did not differ from group housing, suggesting that the short lifespan is not because of a captive artifact or social stress. However, individually housed MZM-0403 lived almost twice as long as group-housed MZM-0403 (maximum lifespan: 53 w vs. 29 w). The mechanism underlying this lifespan difference is currently not clear. It is conceivable that absence of social stress, which is normally induced by male competition, as well as absence of reproductive stress could increase lifespan in both strains but that the high degree of inbreeding in GRZ (see below) might prevent that GRZ benefit from it. This is supported by observations in another recently wild-derived, long-lived *N. furzeri* strain, MZM-0410. The median lifespan of single-housed fish was increased by 10% compared with group-housed fish (unpublished data). We further showed that there are no sex-related lifespan differences in single-housed GRZ and MZM-0403, which is in line with previous reports for group housing (Valenzano *et al.*, 2006b).

Postmortem analysis of GRZ, MZM-0403, and MZM-0410 excluded the failure of a single organ as the cause of death (Di Cicco *et al.*, 2011). Various degenerative lesions were observed in liver, kidney, heart, and gonads, suggesting that death was caused by systemic failure. Similar aging-related lesions were described in other fish species, for example, tubule dilation in the kidney in poeciliid fish (Woodhead *et al.*, 1983) and liver apoptosis in medaka (Ding *et al.*, 2010). Notably, *N. furzeri* showed a marked aging-dependent increase in neoplasias, which was not reported in other fish models. Therefore, the short lifespan of *N. furzeri* might be due to a genuine acceleration of developmental and/or aging processes.

### Genetic structure of *Nothobranchius furzeri* strains GRZ and MZM-0403

This study extends previous reports and, based on a comprehensive marker set, proves that GRZ are highly inbred, whereas MZM-0403 are highly heterogeneous. The data reflect well the origin of the strains, that is, GRZ fish are direct descendants of fish captured in 1968 and have been bred in captivity for at least 80 generations, whereas MZM-0403 fish originate from fish collected in 2004 and have been bred for six generations. In MZM-0403, 35% of the gene-associated markers and 99% of the microsatellite loci were polymorphic; the latter showed an average heterozygosity of 0.51. Similar microsatellite variation estimates were reported for freshwater fish populations (DeWoody & Avise, 2000), suggesting that the genetic composition of MZM-0403 still resembled that of a wild population. By contrast, all markers were monomorphic in GRZ, except for two markers in males. The latter mapped in the sex-linked region, which in our genetic map was located on LG 1. This corresponds well with the XX/XY sex-determination system of *N. furzeri*, in which males are the heterogametic sex (Valenzano *et al.*, 2009).

### Short lifespan and inbreeding depression in the GRZ strain

In spite of genetic homogeneity, the extremely short lifespan of GRZ is likely not due to inbreeding depression only. First, a similarly short lifespan was reported for founders of the GRZ strain (Foersch, 1975). Second, recent studies argue for accelerated aging in GRZ, for example, GRZ showed faster expression of aging-related markers than MZM-0403 (Terzibasi *et al.*, 2008) and earlier onset of aging-dependent neoplasias compared with MZM-0403 (Di Cicco *et al.*, 2011). Third, F_1_ progeny of our cross AB had an intermediate lifespan compared with both GRZ and MZM-0403, which excludes dominant effects of few genes on lifespan. This is in contrast to studies in inbred mice, in which greater heterozygosity was associated with a longer lifespan, for example, F_1_ progeny outlived their parents (Yunis *et al.*, 1984; Gelman *et al.*, 1988). To assess the effect of inbreeding depression in the GRZ strain, one should conduct new and comprehensive samplings in the Gona-Re-Zhou game reserve and/or other semiarid habitats and subsequently analyze lifespan and genetic variability of captive populations and strains.

On the other hand, the lifespan of F_2_ progeny of cross AB was shifted toward the long-lived MZM-0403 phenotype. Why lifespan is shifted this way cannot be elucidated at this stage, but it seems consistent with some compensation of inbreeding depression. It seems further conceivable that the lifespan of replicative crosses will differ with respect to ours because of the heterogeneity of the MZM-0403 strain. The allele set of an MZM-0403 male, which together with the allele set of a GRZ female will be passed onto filial generations, will affect the lifespan of progeny specifically and potentially in a different way compared with another MZM-0403 male.

### An improved genetic map of *Nothobranchius furzeri*

Our second-generation linkage map is based on 355 markers, 65% of which are gene associated and facilitate cross-species analyses. The map covers *c.* 80% of the *N. furzeri* genome. There are currently three more LGs than the number of *N. furzeri* chromosomes; however, the surplus LGs contain no more than three markers, and our calculations allowing large intermarker distances suggest that these LGs will merge with currently distinct LGs (Data S2). The estimated total map length is 2545 cM, that is, roughly two times larger than in medaka (Kimura *et al.*, 2005). This is in good agreement with genome size estimations based on DNA content, suggesting a genome size of 1 Gb for medaka (Kasahara *et al.*, 2007) and 1.6–1.9 Gb for *N. furzeri* (Reichwald *et al.*, 2009). By comparison of gene-associated markers, we found synteny of 19 *N. furzeri* LGs to 22 of 24 medaka Chrs, which corresponds with the number of *N. furzeri* Chrs observed in cytogenetic analyses (Reichwald *et al.*, 2009).

### Mapping of lifespan QTLs

Our analysis revealed that lifespan determination in *N. furzeri* is polygenic. We detected four QTLs affecting lifespan, which were highly significant (LG 9) or suggestive (LG 11, 14 and 17) at the genome-wide level. Taken together, the QTLs explain 27.4% of the total lifespan variance in the F_2_ population. Mapped genetic effects differ in their effects on median and maximum lifespan. In particular, the highly significant QTL increases the mean lifespan by *c.* 18%, but the maximum lifespan only by *c.* 9%, whereas the suggestive QTLs increase median and maximum lifespan to a comparable extent (Table S5), thus illustrating the complexity of the trait. This number of QTLs and the estimation of genes contributing to lifespan variation are consistent with studies in mice, which suggested that lifespan variation of laboratory mice reflects variation at few loci (e.g., 5–10, Miller *et al.*, 1998; Liao *et al.*, 2010).

The heritability of lifespan in our crossing panel was 32%, again being comparable with mice, in which genetic factors contribute to 20–35% of the lifespan variation (Miller *et al.*, 1998; Liao *et al.*, 2010). To our best knowledge, the mouse is the only other vertebrate species, in which lifespan QTLs have been mapped. However, most mouse QTLs show sex-specific effects or epistatic interactions requiring experimental replication (Curtsinger, 2002). In contrast, the *N. furzeri* lifespan QTLs identified in this work do not show sex-specific effects. On the other hand, the QTLs are based on one experiment, in which the long-lived parent comes from a heterozygous strain. It would be highly desirable to generate a collection of short- and long-lived inbred *N. furzeri* strains to confirm our findings.

### Cross-species analysis of QTLs

At current map resolution, the *N. furzeri* QTLs cover large portions of LGs and really represent a starting point for further analyses. In spite of this and the lack of a genome sequence, an initial characterization of the highly significant QTL on LG 9 was performed based on synteny between the *N. furzeri* and medaka genomes and a set of human orthologs of known aging-related genes. We identified the syntenic region on medaka Chr 5 and assigned six known aging-related genes to this region, thus defining a first list of functional candidates. Targeted mapping of two of these genes, *PCK1* and *RAE1*, placed them, however, outside of the *N. furzeri* QTL, likely excluding a major impact on lifespan and pointing to the limitations of a cross-species approach. On the other hand, our attempts to fine map the LG 9 QTL by assigning *N. furzeri* transcripts to the syntenic region in medaka and subsequent genotyping resulted in a doubling of the marker number and a reduction of the 95% CI to 61% of the original length.

The identification of causative variants has been challenging for QTLs identified in established model systems with reference genome sequences available and will be even more challenging for the small effect QTLs we identified. We set out to refine the QTL intervals by generating a larger F_2_-mapping population and an advanced intercross line and will perform RAD-tag mapping of the cross presented here. The identified intervals can be projected onto other fish species, mice, and humans, and candidate genes be defined, as was demonstrated for several quantitative traits in the Mexican cavefish (e.g., Gross *et al.*, 2008). Once a small number of candidate genes have been identified, these can be functionally tested by recently established transgenesis methods (Valenzano *et al.*, 2011). To facilitate fine mapping and further analyses of *N. furzeri* QTLs, we have sequenced the genomes of GRZ and MZM-0403 by next-generation technologies. The assembly of a draft genome and construction of a dense variation catalog are underway. This will pave the way to identify loci relevant for lifespan and fully make use of *N. furzeri* as an alternative genetic vertebrate model for age research.

## Experimental procedures

### Fish husbandry

*Nothobranchius furzeri* GRZ and MZM-0403 and progeny of crosses were kept in 5-L tanks in an open circulation system with centralized water filtration. Room temperature was constant at 26°C; tanks were not heated. For breeding, one male and one female were kept together in a 5-L tank. All progeny was reared individually, that is, two fish in a 5-L tank, divided by a vertical plexiglas. Fertilized eggs were collected every other day and kept at 26°C in Danieau’s medium with daily monitoring. Once eyes were recognizable, embryos were transferred to peat moss until ready to hatch. Hatching was performed as described (Genade, 2006). Fry was fed on nauplii of *Artemia* spp. until 3–4 weeks of age. Adults were fed on C*hironomus* spp. twice a day. Water changes were performed every other day.

### Experimental crosses

In cross A, one GRZ female was crossed with one MZM-0403 male. Of the F_1_ progeny (*n* = 9), three fish (one male, two females) were intercrossed and gave rise to 39 F_2_. In cross B, another GRZ female and MZM-0403 male were crossed. Of the F_1_ progeny (*n* = 15), six breeder pairs (one male, one female each) were formed. Two males were used in two F_1_ families, that is, ten (six females, four males) F_1_ fish were interbred and produced 365 F_2_ offspring. Eggs were collected every other day and kept separately for each cross/breeder pair.

### Recording of phenotypes

Sex, lifespan (age at death), and body weight were recorded. All numbers refer to fish, which we successfully genotyped. Sex determination relied on the presence of caudal fin coloration at 6 weeks of age; sex could be determined for 404 F_2_ (i.e., all F_2_ of cross A and B). The sex ratio was assumed to be 1:1 in F_2_ progeny and tested using the chi-square test (SigmaPlot 11; Systat Software, Inc., Erkrath, Germany). The sex (male to female) ratio of the F_2_ populations was even, that is, 19:20 in cross A and 182:183 in cross B.

Tanks were checked twice a day for dead fish. If dead, fish were immediately frozen at −20°C. If death was not natural, for example, caused by a broken water supply, fish were excluded from analysis. Lifespan could be recorded for all F_1_ and 27 F_2_ of cross A and 283 F_2_ of cross B.

Body weight was recorded for 286 F_2_ progeny of cross B from 4 weeks of age, that is, once sexual maturity was reached, and males/females could be identified. Recordings were carried out once every other week using a laboratory precision balance. Maximal body weight refers to maximal weight ever reached during lifetime (Data S3).

### Sequencing

We performed genomic sequencing of *N. furzeri* strains GRZ, MZM-0403, and MZM-0410 and one male specimen from the Mazimechopes River, Mozambique, kindly provided by M. Schartl as reported (Reichwald *et al.*, 2009); we generated a total of 14.74, 5.81, 0.7, and 0.79 Mb, respectively. Using Sanger technology, we further sequenced a normalized cDNA library (Evrogen, Moscow, Russia) prepared from whole body of one male, 9-week-old GRZ. Sequences of 7101 genomic (5.64 Mb) and 8377 cDNA clones (7.68 Mb) were used for blast analyses as outlined in marker identification. Resequencing of candidate genes was carried out using Sanger technology (Data S4).

### Marker identification and genotyping

To establish SNVs, a two-step blast search was performed (Data S4). Significant hits (*P* < 10^−10^) were found for 1320 sequences (cDNA and genomic). Further, 16 *N. furzeri* orthologs of aging-relevant genes were identified and cloned as described (Hartmann *et al.*, 2009; Reichwald *et al.*, 2009). Primer pairs were designed for all gene fragments using the GAP4 module of the Staden Sequence Analysis Package (Staden, 1996) and ordered from Metabion (Martinsried, Germany). PCRs were performed for all DNA fragments, 801 amplicons were obtained and analyzed, and 324 informative SNVs and 128 informative microsatellites were identified and genotyped as outlined in Data S4.

### Building a second-generation linkage map

Four P_0_-, 13 F_1_-, and 404 F_2_ specimens of cross AB were genotyped at those 411 markers, for which all alleles were strain specific in the P_0_. Success rate was 99% for microsatellites and 85% for SNVs. Genotypes of 368 markers, including 240 SNVs, were used for map calculation. Linkage distance and marker order were calculated by two-point analysis using mapmaker/exp 3.0b for Unix (http://www.nslij-genetics.org/soft/mapmaker), the Kosambi mapping function, a maximum intermarker distance of 36 cM, and a minimum LOD score of 4.0. The marker order along LGs was determined based on maximum likelihood scores. To calculate the map length, two methods were used and the average of both estimates given as previously proposed (Tripathi *et al.*, 2009). Linkage groups were numbered according to genetic length (longest LG = LG 1) and drawn using MapChart 2.2 (Voorrips, 2002).

### Statistical analyses

Calculations were performed using SigmaPlot 11 (Systat Software, Inc., 2008). Minimum, maximum, mean, and median trait values as well as standard deviation and variance were determined. Lifespan data were tested for normal distribution using the Kolmogorov–Smirnov test. For the first QTL analysis, the entire F_2_ population of cross B was used. Square-root-transformed lifespan values were used because raw data were not normally distributed (*P* = 0.338); square-root-transformed data showed normal distribution. We tested for an influence of sex by using the Mann–Whitney test, and for an influence of breeder family using the Kruskal–Wallis test (one-way anova); there was no significant effect (sex: *P* = 0.087; breeder family: *P* = 0.319). However, maximal body weight and lifespan were correlated (*R* = 0.319, *P* < 0.001, Spearman’s rank correlation). Experiment-specific significance levels were determined by permutation analysis (Table S8). Prior to the second QTL analysis, 25% of F_2_ progeny (*n* = 71), which died early, were excluded. The lifespan data of the remaining F_2_ specimens were transformed into integer numbers (rank order), from 1 (the first fish, which died after censoring the 25% of early deaths) to 213 for the longest-lived fish. Ranks were then transformed into a Gaussian distribution by assigning them into nine classes (Table S9). Classes showed a normal distribution (mean = 5.0, standard deviation = 1.40). Sex and maximum body weight did not affect ranked lifespan data (sex: *P* = 0.733, maximal body weight: *P* = 0.097).

Log-rank statistic was used to analyze survivorship of parental strains and cross B.

### QTL analysis

QTLs were mapped using GridQTL (Hernandez-Sanchez *et al.*, 2009); for details on the approach see (Haley & Knott, 1992). On the basis of statistical analyses, the standard model to identify lifespan QTLs explaining the phenotypic variance in the F_2_ generation included maximum body weight as covariate in the first QTL scan and did not include covariates in the second QTL scan. Genome-wide QTL scans were performed using the forward selection interval mapping approach (Carlborg *et al.*, 2005). First, the standard interval mapping model, which assumes a single QTL on a given Chr or LG, including both additive and dominance effects, was fitted to the data. It was tested whether a model of a single QTL affecting the trait was superior to a model of no QTL; the interval size was 1 cM. If the calculated *F*-test statistic exceeded a specific threshold, a single-trait QTL was inferred to be present at the position showing the highest statistical value. Next, a scan was performed in which the previously found most significant QTL was added as cofactor to the model. This procedure was repeated until no further significant QTL was detected. Finally, the location of each significant QTL was revised by testing exclusively the LG on which it was located while regarding all other significant QTLs as genetic background effects. Estimated QTL positions are given as centimorgan distance from one randomly chosen telomeric end for each LG.

Empirically derived significance thresholds for one-QTL vs. no-QTL test statistics were estimated using the permutation test (Churchill & Doerge, 1994); 1000 permutations of the data were analyzed as for the unpermutated data. The following thresholds were derived: genome-wide highly significant (*P* = 0.01) and significant (*P* = 0.05) and LG-wise highly significant (*P* = 0.01) and significant (*P* = 0.05). To the best of our knowledge, a Chr-wide threshold of *P* = 0.05 corresponds to a genome-wide suggestive threshold of *P* = 0.63 in the mouse (e.g., Brockmann *et al.*, 1998). This estimation is based on the number of Chr. Because *N. furzeri* has approximately the same number of Chr as the mouse, it is reasonable to apply the LG-wise threshold of *P* = 0.05 as genome-wide ‘suggestive’ threshold. A parametric bootstrap with 1000 iterations was performed to estimate the 95% CI of a single QTL (Visscher *et al.*, 1996).

### Estimation of lifespan heritability and gene number

Heritability was calculated using the formula H^2^ = V_G_/V_P_, where V_G_ is the genetic variance and V_P_ the phenotypic variance of different populations. The number of genes influencing lifespan was estimated using the Castle–Wright estimator (Castle, 1921).

### Assigning of human aging-relevant genes to medaka

Nucleotide sequences of 261 human orthologs of aging-relevant genes were downloaded from ‘The Aging Gene Database’. The location of their orthologs in medaka was determined by blat (UCSC Genome Browser, Medaka Oct. 2005 version 1 draft assembly, oryLat2).

### Mapping of *Nothobranchius furzeri* markers in medaka

The 355 *N. furzeri* markers (average length of sequences: 1219 ± 476 nt) contained in our genetic map were searched using blastn against the medaka genome (version as given above); hits with *P* < 10^−8^ were considered significant. Synteny was defined based on the presence of at least two markers on both an *N. furzeri* LG and a medaka Chr.

## Supporting Information

**Fig. S1** Allele distribution of 82 microsatellites in GRZ and MZM-0403.

**Fig. S2** Linkage map.

**Fig. S3** Oxford plot of *N. furzeri* and medaka.

**Fig. S4** Lifespan of cross B.

**Fig. S5** First QTL scan.

**Table S1** Lifespan of F_2_ males sorted by color

**Table S2** Details of the linkage map

**Table S3** Pedigree of cross B

**Table S4** Results of the second QTL scan

**Table S5** Detailed analyses of QTLs

**Table S6** All markers on LG 9 including the markers identified after fine-mapping

**Table S7** Results of the QTL scan after fine mapping of LG 9

**Table S8** Results of the first QTL scan

**Table S9** Ranked data

**Data S1** LG 1 markers showing sex-biased alleles

**Data S2** Male and female linkage map

**Data S3** Body weight data

**Data S4** Marker identification, validation and genotyping

As a service to our authors and readers, this journal provides supporting information supplied by the authors. Such materials are peer-reviewed and may be re-organized for online delivery, but are not copy-edited or typeset. Technical support issues arising from supporting information (other than missing files) should be addressed to the authors.
