# Supplementary figures and images for: Mapping of quantitative trait loci controlling lifespan in the short-lived fish Nothobranchius furzeri – a new vertebrate model for age research
Source: Aging Cell. 2012 Apr;11(2):252–61. doi: 10.1111/j.1474-9726.2011.00780.x (PMC3437503; doi:10.1111/j.1474-9726.2011.00780.x)

## Slide 1
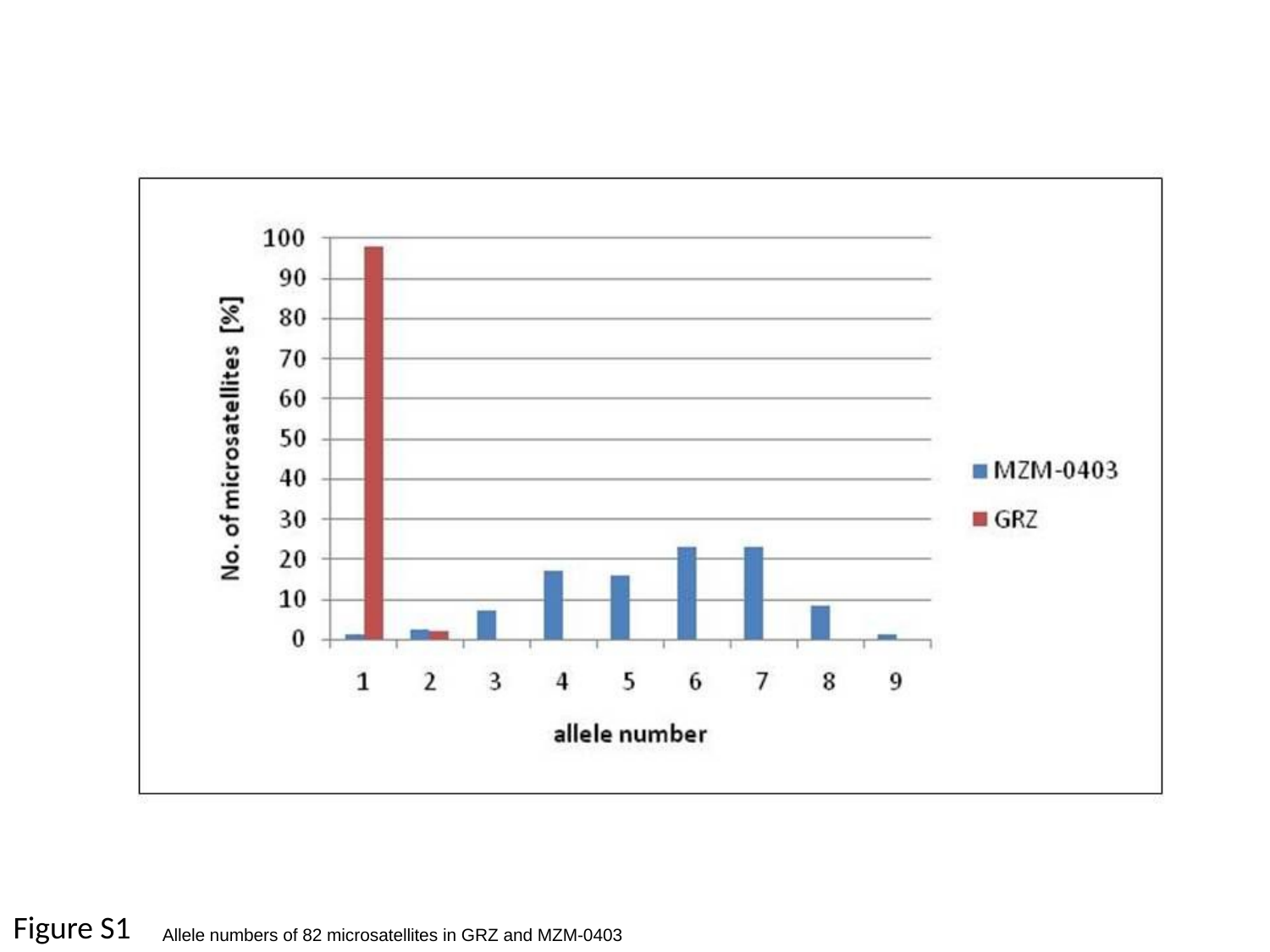

Figure S1
Allele numbers of 82 microsatellites in GRZ and MZM-0403

Supplement: Supplementary file 1 [file acel0011-0252-SD10.pptx]
