# Supplementary material for: Mapping of quantitative trait loci controlling lifespan in the short-lived fish Nothobranchius furzeri – a new vertebrate model for age research
Source: Aging Cell. 2012 Apr;11(2):252–61. doi: 10.1111/j.1474-9726.2011.00780.x (PMC3437503; doi:10.1111/j.1474-9726.2011.00780.x)
Supplement: Supplementary file 2 [file acel0011-0252-SD11.pptx]

## Slide 1
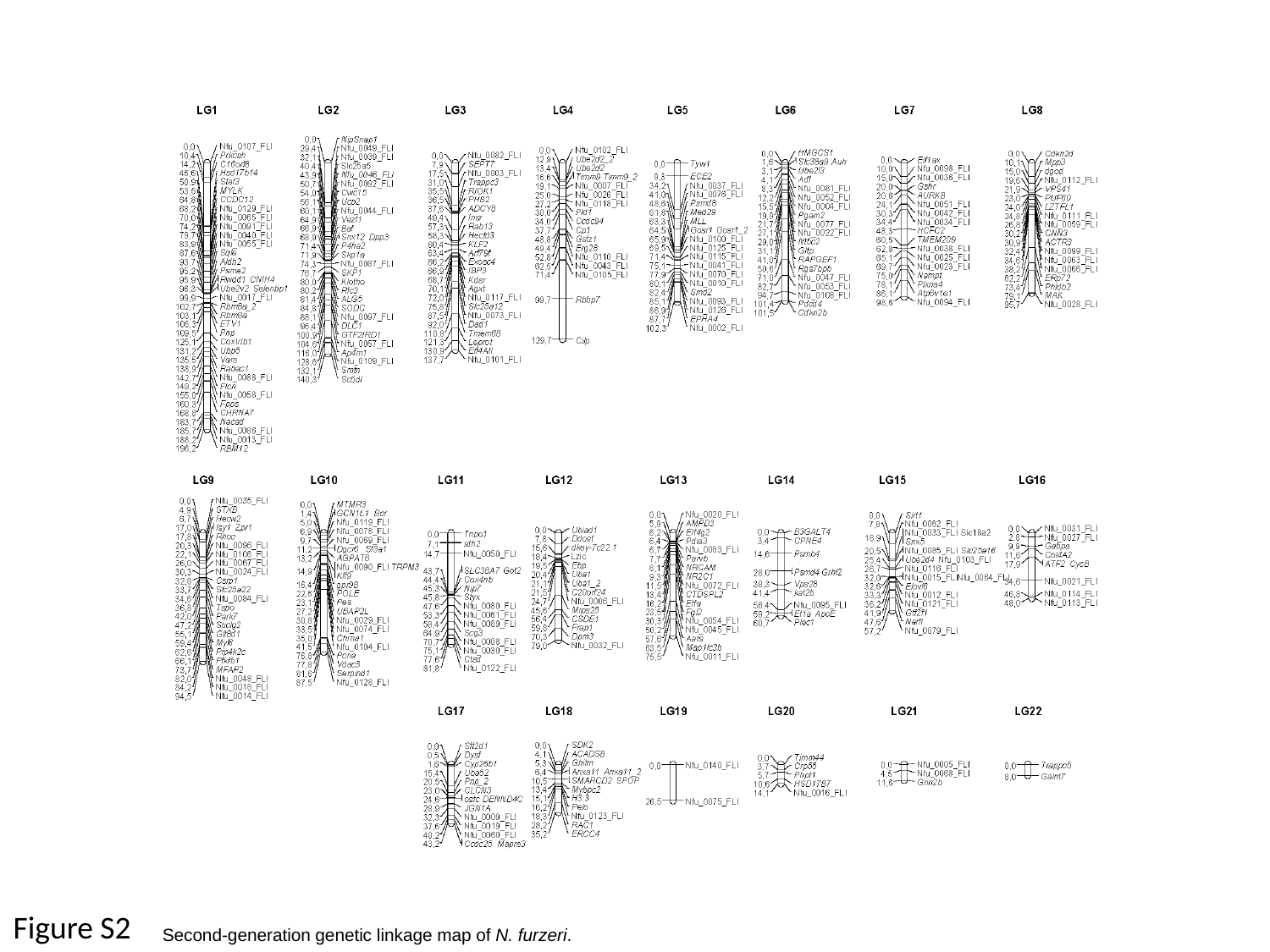

Figure S2
Second-generation genetic linkage map of N. furzeri.

## Slide 2
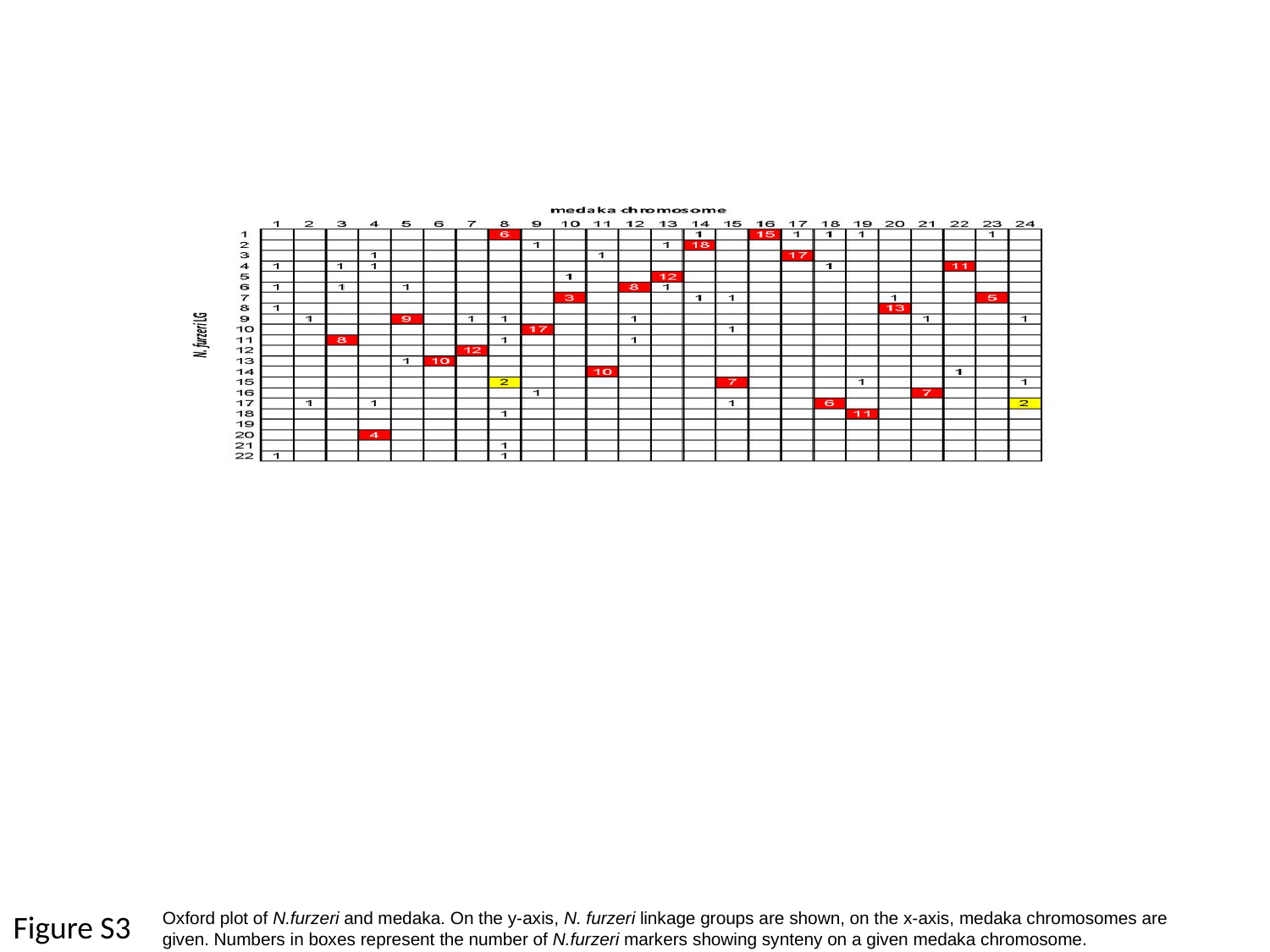

Figure S3
Oxford plot of N.furzeri and medaka. On the y-axis, N. furzeri linkage groups are shown, on the x-axis, medaka chromosomes are given. Numbers in boxes represent the number of N.furzeri markers showing synteny on a given medaka chromosome.

## Slide 3
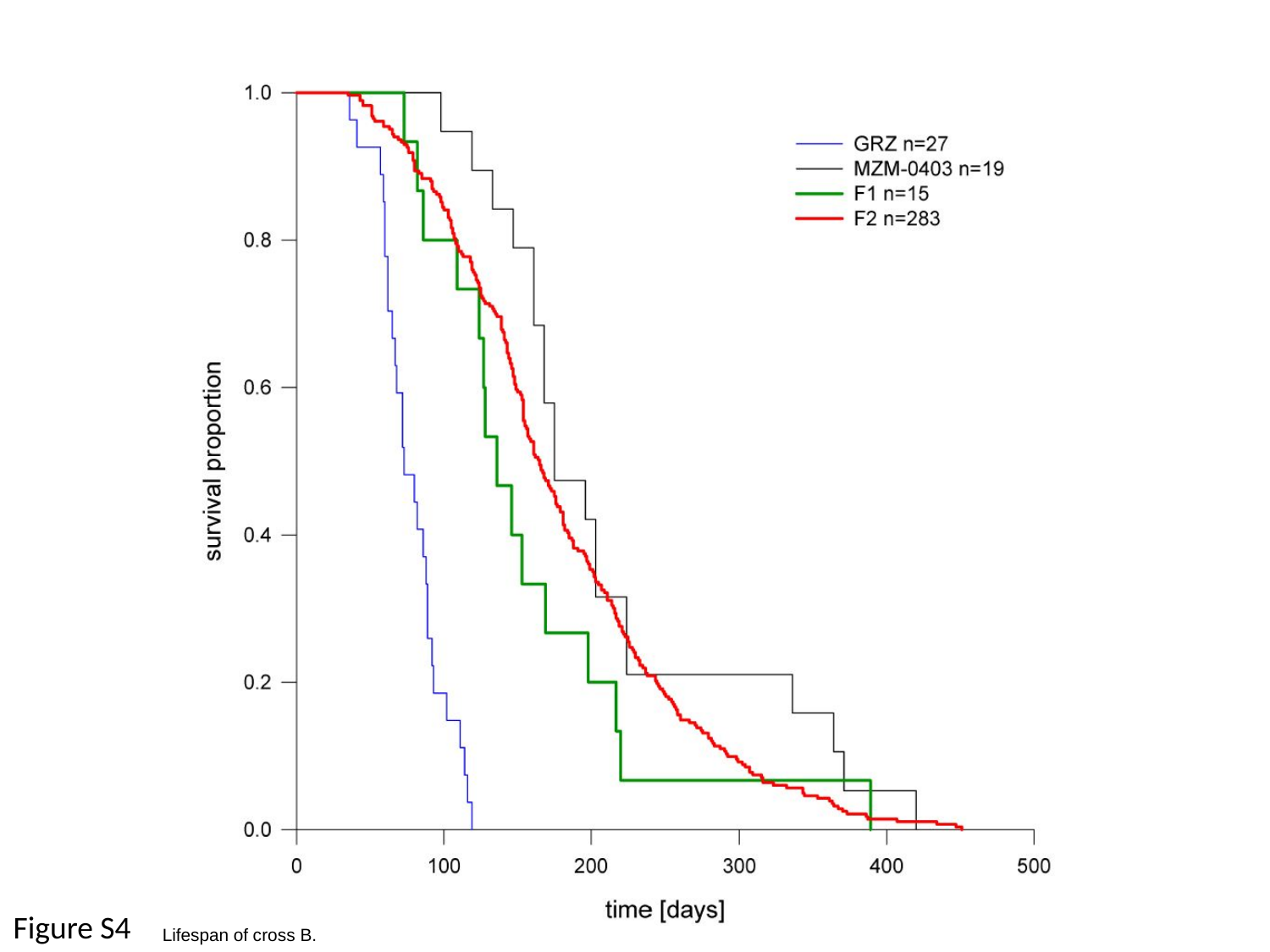

Figure S4
Lifespan of cross B.

## Slide 4
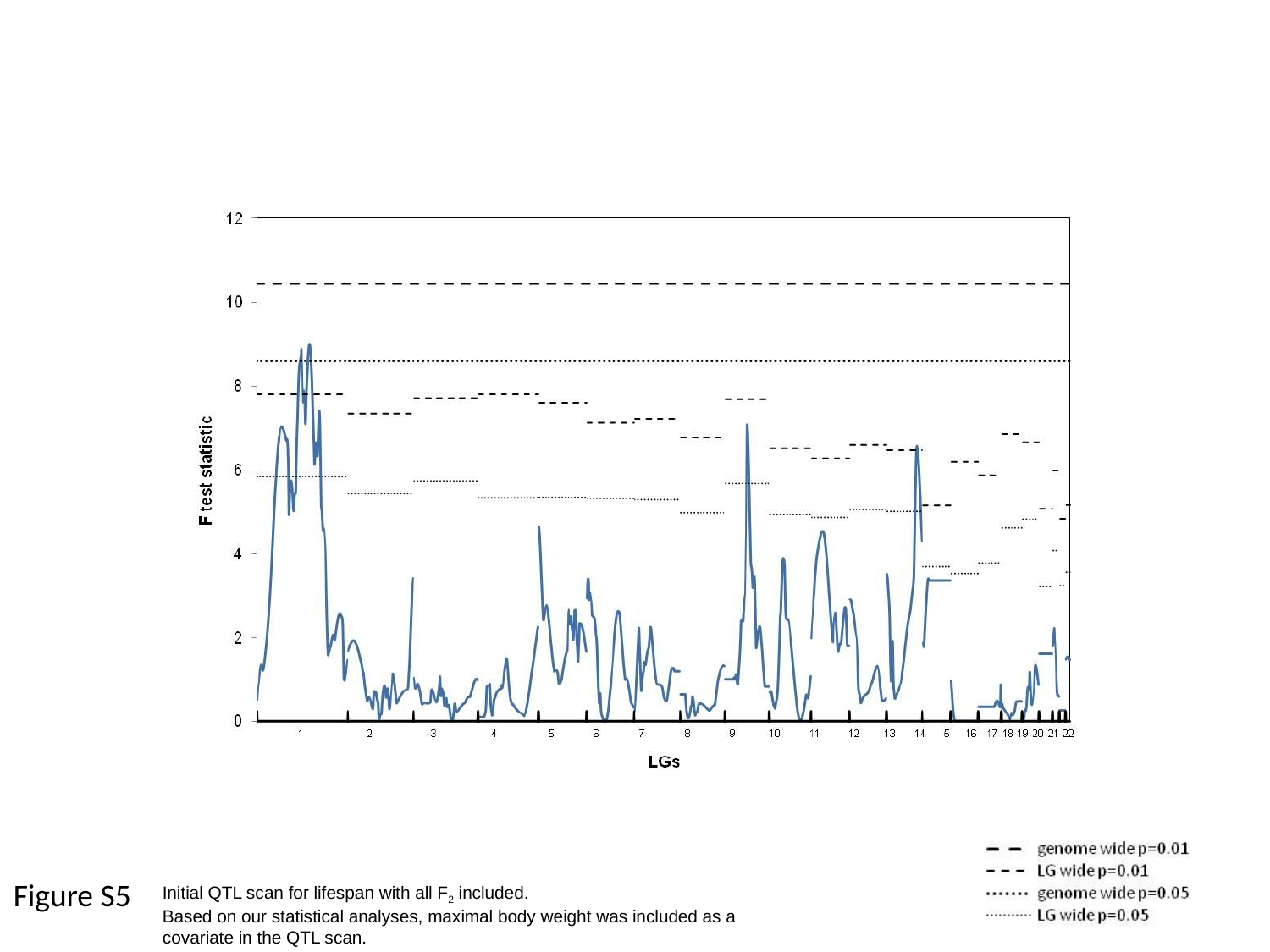

Figure S5
Initial QTL scan for lifespan with all F2 included.
Based on our statistical analyses, maximal body weight was included as a covariate in the QTL scan.
