# Supplementary material for: Mapping of quantitative trait loci controlling lifespan in the short-lived fish Nothobranchius furzeri – a new vertebrate model for age research
Source: Aging Cell. 2012 Apr;11(2):252–61. doi: 10.1111/j.1474-9726.2011.00780.x (PMC3437503; doi:10.1111/j.1474-9726.2011.00780.x)
Supplement: Supplementary file 4 [file acel0011-0252-SD2.doc]

## Supplementary Table 2: Second-generation genetic linkage map of N.furzeri

| **A) Summary** |  |
| --- | --- |
| Number of specimens | 404 |
| Number of linkage groups | 22 |
| Number of linked markers | 355 |
| Number of singletons | 13 |
| Number of genetically separated marker pairs | 309 |
| Length of map | 1,969 cM |
| Average length/linkage group (range of length) | 90 (21 - 207) cM |
| Average marker number per linkage group | 16 (2 - 37) |
| Average marker distance | 5.5 cM |
| Maximal marker distance | 35.3 cM (LG 10) |
| Average LOD score | 86 |
| Maximal LOD score | 162 |
| Average recombination frequency | 0.050 |
| Physical distance | 0.63‑0.75 Mb/1cM |

| **B) Length of Linkage groups and number of markers** | | |
| --- | --- | --- |
| LG | Number of markers | Length (cM)* |
| 1 | 37 | 206.6 |
| 2 | 29 | 150.2 |
| 3 | 24 | 148.6 |
| 4 | 18 | 142.2 |
| 5 | 20 | 112.6 |
| 6 | 20 | 111.7 |
| 7 | 17 | 109.7 |
| 8 | 18 | 106.2 |
| 9 | 24 | 103.5 |
| 10 | 24 | 96.2 |
| 11 | 16 | 92.2 |
| 12 | 14 | 90.0 |
| 13 | 17 | 85.1 |
| 14 | 11 | 71.7 |
| 15 | 18 | 65.5 |
| 16 | 9 | 58.9 |
| 17 | 14 | 51.4 |
| 18 | 13 | 43.0 |
| 19 | 2 | 57.9 |
| 20 | 5 | 22.5 |
| 21 | 3 | 22.3 |
| 22 | 2 | 20.9 |
| Total | 355 | 1,968.9 |

* calculated according to (Tripat*hi et a*l. 2009)
