# Supplementary material for: Mapping of quantitative trait loci controlling lifespan in the short-lived fish Nothobranchius furzeri – a new vertebrate model for age research
Source: Aging Cell. 2012 Apr;11(2):252–61. doi: 10.1111/j.1474-9726.2011.00780.x (PMC3437503; doi:10.1111/j.1474-9726.2011.00780.x)
Supplement: Supplementary file 5 [file acel0011-0252-SD3.doc]

**Supplementary Table 3: F_2_ pedigree structure of cross B**

| **F_1_ breeding family** | **Number of offspring^1^** | **Number of offspring**  **with lifespan data^1^** |
| --- | --- | --- |
| ♀1 x ♂1 | 34 (20; 14) | 009 (5; 4) |
| ♀2 x ♂2 | 7 (4;3) | 005 (3, 2) |
| ♀3 x ♂3 | 60 (33; 27) | 037 (19; 18) |
| ♀4 x ♂3 | 62 (30; 32) | 047 (23; 24) |
| ♀5 x ♂4 | 3 (0; 3) | 005 (0; 2) |
| ♀6 x ♂4 | 199 (95; 104) | 184 (86; 98) |
| **Total:** 10 (4;6) | 365 (182; 183) | 284 (136; 148) |

^1^ in parenthesis: (male; female)
