# Supplementary material for: Mapping of quantitative trait loci controlling lifespan in the short-lived fish Nothobranchius furzeri – a new vertebrate model for age research
Source: Aging Cell. 2012 Apr;11(2):252–61. doi: 10.1111/j.1474-9726.2011.00780.x (PMC3437503; doi:10.1111/j.1474-9726.2011.00780.x)
Supplement: Supplementary file 12 [file acel0011-0252-SD12.doc]

## Supplementary Dataset 1 – First genome-wide QTL analysis

1. Sex bias in allele distribution of markers at LG 1

A more detailed analysis of the QTL on LG 1, which was detected in the initial QTL scan, revealed that the 95% confidence interval comprised 30 markers, all of which showed a sex-biased allele distribution. This was most obvious at microsatellite loci, at which one allele from the male P_0_ parent was almost exclusively found in males and almost absent in females (Table 1).

|  | **male allele 1 (%)** | | **male allele 2 (%)** | | **female allele (%)** | |
| --- | --- | --- | --- | --- | --- | --- |
| **markers** | male | female | male | female | male | female |
| Nfu_0129_FLI | 44 | 1 | 28 | 27 | 28 | 72 |
| Nfu_0065_FLI | 43 | 1 | 29 | 27 | 28 | 73 |
| Nfu_0091_FLI | 43 | 1 | 37 | 26 | 20 | 74 |
| Nfu_0040_FLI | 41 | 1 | 25 | 25 | 34 | 74 |
| Nfu_0055_FLI | 42 | 1 | 27 | 26 | 31 | 73 |
| Nfu_0017_FLI | 43 | 1 | 26 | 25 | 31 | 74 |
| Nfu_0088_FLI | 11 | 5 | 55 | 31 | 33 | 63 |
| Nfu_0058_FLI | 38 | 29 | 24 | 13 | 39 | 58 |
| Nfu_0086_FLI | 13 | 8 | 38 | 43 | 49 | 48 |

Table 1: Allele distribution of microsatellite markers locates in the 95% confidence interval of the lifespan QTL at LG 1, as identified in the intital QTL scan including all F_2_ progeny. The descriptors “male” and “female” in respective columns refer to F_2_ males (n=136) and females (n=148).
