# Supplementary material for: Mapping of quantitative trait loci controlling lifespan in the short-lived fish Nothobranchius furzeri – a new vertebrate model for age research
Source: Aging Cell. 2012 Apr;11(2):252–61. doi: 10.1111/j.1474-9726.2011.00780.x (PMC3437503; doi:10.1111/j.1474-9726.2011.00780.x)
Supplement: Supplementary file 13 [file acel0011-0252-SD13.doc]

## Supplementary Dataset 2 – Details of the genetic map

We first built a male and a female map, which we found consistent in marker order, map length and average recombination frequency (female: 0.045, male: 0.048; ratio 1 : 1.07) (see Table 1 and 2 below). This was different from the expectation, because in many fish species females show higher recombination rates than males (e.g. Sing*er et a*l. 2002; Ghar*bi et a*l. 2006; Re*id et a*l. 2007; Rexro*ad et a*l. 2008). In the sex-averaged second-generation genetic map of *N. furzeri*, the largest LG (LG 1) was 206.6 cM, the smallest LG (LG 22) was 20.9 cM and the average LG length was 90 cM. Further, there were on average sixteen markers per LG. Most (218/355, 66%) inter-marker intervals were less than 5 cM, 20% (67) ranged from 5 cM to 10 cM, 11% (37) were between 10‑20 cM, and 3% (11) were larger than 20 cM. The three surplus LGs (LG 19, 21 and 22) contained three markers at most.

Seven marker pairs of the current genetic map show inter-marker distances larger than 25 cM and are located on six LGs. These marker pairs are given below (sorted by ascending inter-marker distance, listed in the following way: marker 1 – marker 2, inter-marker distance, LOD score as estimated by MAPMAKER/EXP 3.0b):

1] LG 19: Nfu_0140_FLI ‑ Nfu_0075_FLI, 26.5 cM, LOD 4.22

2] LG 4: Nfu_0105_FLI *‑ Rbbp7‑Clip*, 28.3 cM, LOD 25.68; 30.0 cM, LOD 25.62

3] LG 11: Nfu_0050_FLI *‑ Got2/ Scl38a7*, 29.0 cM, LOD 22.24

4] LG 2: *NipSnap1* ‑ Nfu_0049_FLI, 29.4 cM, LOD 20.19

5] LG 1: *C16orf8 ‑ Hsd17b14*, 32.4 cM, LOD 5.19

6] LG 10: Nfu_0104_FLI *‑ Pcna*, 35.3 cM, LOD 12.29

It is likely that the three LGs formed by two or three markers (LG 19, 21 and 22) will coalesce with presently distinct LGs once more markers/meiosis will have been analyzed. For example, if we performed a map calculation by allowing a relaxed inter-marker distance (> 36 cM < 50 cM), LG 19 joined to LG 7 (via gene-associated marker *Eif1ax* of LG 7 and microsatellite marker Nfu_0075_FLI of LG 19, inter-marker distance 46.6 cM, LOD 4.35). Further, by BLASTN comparison (as outlined in “experimental procedures” in the main manuscript) of the above *N. furzeri* marker pairs against the genome of medaka, we found that three marker pairs showed synteny. These were marker pairs on *N. furzeri* LG 1, 4 and 11 showing the same order on medaka chromosome 8, 22 and 3, respectively .

**Table 1: Genetic linkage map, calculated for female F_2_** of cross AB

| **Linkage group** | **Number of markers** | **Length [cM]** |
| --- | --- | --- |
| 01 | 28 | 148,7 |
| 02 | 18 | 144,1 |
| 03 | 24 | 137,6 |
| 04 | 31 | 133,2 |
| 05 | 20 | 111,0 |
| 06 | 20 | 108,6 |
| 07 | 15 | 104,8 |
| 08 | 17 | 89,4 |
| 09 | 15 | 86,4 |
| 10 | 14 | 81,3 |
| 11 | 19 | 74,7 |
| 12 | 17 | 74,9 |
| 13 | 13 | 51,1 |
| 14 | 20 | 48,9 |
| 15 | 12 | 41,5 |
| 16 | 15 | 29,6 |
| 17 | 5 | 33,0 |
| 18 | 3 | 26,0 |
| 19 | 3 | 24,9 |
| 20 | 5 | 19,3 |
| 21 | 6 | 18,1 |
| 22 | 4 | 15,4 |
| 23 | 2 | 20,2 |
| 24 | 2 | 20,2 |
| 25 | 4 | 12,5 |
| 26 | 3 | 13,5 |
| 27 | 3 | 12,2 |
| 28 | 2 | 11,0 |
| 29 | 2 | 4,2 |
| **total** | **342** | **1696,1** |

**Table 2: Genetic linkage map, calculated for male F_2_** of cross AB

| **Linkage group** | **Number of markers** | **Length [cM]** |
| --- | --- | --- |
| 01 | 24 | 155,8 |
| 02 | 33 | 145,8 |
| 03 | 28 | 142,4 |
| 04 | 16 | 121,0 |
| 05 | 20 | 113,5 |
| 06 | 19 | 113,4 |
| 07 | 24 | 102,1 |
| 08 | 14 | 104,4 |
| 09 | 13 | 99,2 |
| 10 | 24 | 91,6 |
| 11 | 16 | 93,5 |
| 12 | 16 | 92,9 |
| 13 | 16 | 92,6 |
| 14 | 13 | 43,5 |
| 15 | 14 | 42,8 |
| 16 | 7 | 41,6 |
| 17 | 15 | 32,9 |
| 18 | 5 | 24,5 |
| 19 | 3 | 26,2 |
| 20 | 4 | 20,3 |
| 21 | 3 | 21,7 |
| 22 | 2 | 20,6 |
| 23 | 2 | 15,4 |
| 24 | 4 | 8,3 |
| 25 | 2 | 4,6 |
| **total** | **337** | **1770,6** |

References:

Gharbi K, Gautier A, Danzmann RG, Gharbi S, Sakamoto T, Hoyheim B, Taggart JB, Cairney M, Powell R, Krieg F, Okamoto N, Ferguson MM, Holm LE, Guyomard R (2006). A linkage map for brown trout (Salmo trutta): chromosome homeologies and comparative genome organization with other salmonid fish. *Genetics*. **172**, 2405-2419.

Singer A, Perlman H, Yan Y, Walker C, Corley-Smith G, Brandhorst B, Postlethwait J (2002). Sex-specific recombination rates in zebrafish (Danio rerio). *Genetics*. **160**, 649-657.

Reid DP, Smith CA, Rommens M, Blanchard B, Martin-Robichaud D, Reith M (2007). A Genetic linkage map of Atlantic halibut (Hippoglossus hippoglossus L.). *Genetics*. **177**, 1193-1205.

Rexroad CE, 3rd, Palti Y, Gahr SA, Vallejo RL (2008). A second generation genetic map for rainbow trout (Oncorhynchus mykiss). *BMC Genet*. **9**, 74.
