# Supplementary material for: Mapping of quantitative trait loci controlling lifespan in the short-lived fish Nothobranchius furzeri – a new vertebrate model for age research
Source: Aging Cell. 2012 Apr;11(2):252–61. doi: 10.1111/j.1474-9726.2011.00780.x (PMC3437503; doi:10.1111/j.1474-9726.2011.00780.x)
Supplement: Supplementary file 14 [file acel0011-0252-SD14.doc]

## Supplementary Dataset 3 – Body weight recordings

We recorded body weight in the F_2_ progeny of cross B because we had previously observed that *N. furzeri* males tended to be bigger than females and that the fish grow/ get heavier throughout life (unpublished). A significant weight difference between males and females was detected already at the onset of sexual maturation (week 4-5) and persisted throughout life. As we expected, males weighed more than females (Figure 1). The mean body weight of males in week 4 was 0.8 ± 0.4 g (range: 0.2 – 2.1 g), whereas females weighed 0.5 ± 0.3 g (range: 0.1 – 1.9 g). The weight difference was found to be highest at old age, i.e. mean maximal body weight for males was 3.8 ± 1.1 g (range: 0.5 – 6.7 g) and 2.2 ± 0.6 g (range: 0.2 ‑  3.6 g) for females (all *P*<0.001, Mann-Whitney test).

Figure 1: The body weight of cross B, recorded once every other week using a laboratory balance. Red circles show the mean of females (n=148), blue circles depict the mean of males (n=138), and green circles represent the mean of both sexes (n=286).
