# Supplementary material for: Mapping of quantitative trait loci controlling lifespan in the short-lived fish Nothobranchius furzeri – a new vertebrate model for age research
Source: Aging Cell. 2012 Apr;11(2):252–61. doi: 10.1111/j.1474-9726.2011.00780.x (PMC3437503; doi:10.1111/j.1474-9726.2011.00780.x)
Supplement: Supplementary file 15 [file acel0011-0252-SD15.doc]

## Supplementary Dataset 4 – Marker identification and experimental analysis

## Marker identification

To establish SNVs, BLASTX searches using genomic and cDNA sequences as queries against Swiss‑Prot/TrEMBL (version 50.6) were performed to identify gene-associated sequences. Significant hits (*P*<10^-10^) were found for 124 genomic and 1,196 cDNA sequences. These 1,320 sequences were used as queries in a TBLASTX search against cDNA sequences of model fishes. The latter were downloaded from *Ensembl*; i.e. *Tetraodon nigroviridis* update release 2007-05-29, *Gasterosteus aculeatus* update release 2007-05-30, *Oryzias latipes* update release 2007-05-24 and *Danio rerio* update release 2007-07-30. *N. furzeri* orthologs of aging-relevant genes were identified by BLASTX searches in our cDNA sequences and cloned as described (Hartma*nn et a*l. 2009; Reichwa*ld et a*l. 2009). The genes were *N. furzeri* *APOE*, *CDKN2B*, *CDKN2D*, *EXO1*, *FOXO3A*, *GIB1, HPRT*, *IGF1R*, *INSR*, *KLOTHO, MSRA*, *SHC3*, *SIRT1,* *TBP*, *TERT* and *TP53*. Primer pairs were designed in 1,336 gene fragments (124 in genomic DNA, 1,196 in cDNA and 16 in aging-related genes), using the GAP4 module of the Staden Sequence Analysis Package (Staden 1996) and ordered from Metabion (Martinsried, Germany).

## PCR

PCRs were performed for all potenital markers. The PCR setup was: 50 ng DNA, 10 pmol of each primer with one primer fluorescently labeled (6-FAM, Metabion, Martinsried, Germany; NED or VIC, Applera Deutschland GmbH, Darmstadt, Germany) and one PuReTaq Ready-To-Go PCR bead (GE Healthcare, Munich, Germany). The PCR program was: 94°C 5 min, 35 cycles of 94°C 30 sec, 56°C 30 sec and 72°C 1 min, and 72°C 60 min.

## DNA isoalation

*SNVs*. For PCR: Genomic DNA was extracted from 30 mg frozen tissue using the AquaGenomic Kit (MobiTec, Goettingen, Germany). For genotyping of SNVs using the SNPlex™ Genotyping System (Applied Biosystems, Foster City, CA, USA): Genomic DNA was extracted using the DNeasy 96 Blood & Tissue Kit (Qiagen, Hilden, Germany) and at least 30 ng DNA were amplified with the illustra GenomiPhi V2 DNA Amplification Kit (GE Healthcare, UK). *Microsatellites*. Extraction of genomic DNA was done using the AquaGenomic Kit as described for SNVs.

## Genotyping of SNVs

We analyzed 745 SNVs in one GRZ/MZM-0403 specimen and a pool of 10 GRZ/ MZM‑0403 specimens, respectively, and 591 SNVs in the P_0_ of cross AB. 801 amplicons were obtained, sequenced and analyzed. SNVs showing strain-specific alleles (324) were considered informative and found in 313 genes. For 283 informative SNVs (corresponding with 275 genes), genotyping assays using the SNPlex™ Genotyping System were established (Applied Biosystems, Foster City, CA, USA). SNPlex genotyping was done in 417 specimens, i.e. F_1_ breeders and all F_2_ of cross AB. Genotypes of 263 SNVs were obtained, of which 240 SNVs were informative in cross A and B (raw data are available for download on request).

## Genotyping of microsatellites

To analyze the genetic variability of *N. furzeri* strains, 47 microsatellites were genotyped in 10 GRZ (7 males, 3 females) and MZM-0403, each. Another 35 microsatellites were genotyped in 2 GRZ and a pool of another 10 GRZ, as well as in 10 MZM‑0403 and a pool of another 10 MZM‑0403. A further 57 microsatellites were genotyped in P0 of cross A and B.

For genotyping of microsatellites, 2-4 amplicons were pooled and separated on ABI 3730xl capillary sequencers including the GeneScan™ 500 LIZ® Size Standard (Applied Biosystems, Foster City, CA, USA). The fragment length was determined using GeneMapper v4 (Applied Biosystems, Foster City, CA, USA). Allele calling was done independently by two individuals. Discrepancies were resolved by re-genotyping. The resulting 128 informative microsatellites, 126 in cross A and 102 in cross B, were genotyped the same way in P0, F_1_ breeder pairs and F_2_ populations of cross AB. The nomenclature is: a species identifier (Nfu) is followed by a unique, four digit number and a three letter code identifying the institute where the marker was developed, i.e. Nfu_0001_FLI.

## Resequencing *N. furzeri* *SUCLG2* in the P_0_ of cross B, establishing of markers in candidate genes and genotyping

The relevant cDNA sequences were identified by TBLASTX searches of our cDNA data against the non-redundant section of NCBI (http://blast.ncbi.nlm.nih.gov/Blast.cgi). Primers were defined in exons to amplify across exon/intron boundaries. PCRs were performed in genomic DNA of the P0 specimens of cross B. Amplicons were directly sequenced using PCR primers and visually inspected as described above. If necessary, new primers were defined to completely amplify/sequence exons. Informative SNVs were identified by comparison of GRZ to MZM-0403 sequences and genotyped in cross B (F_1_ and F_2_; n=294) by direct sequencing. In *N. furzeri* genes *PCK1*, *RAE1* and *CDC42*, intronic SNVs were genotyped. We note that the SNV in *CDC42* were heterozygous in the male P0; and resequencing of the entire locus in the MZM-0403 male P_0_ revealed only heterozygous variations (data not shown).
